# Phosphatidylinositol 4-kinase IIIβ mediates contraction-induced GLUT4 translocation and shows its anti-diabetic action in cardiomyocytes

**DOI:** 10.1007/s00018-020-03669-7

**Published:** 2020-10-22

**Authors:** A. Sun, O. Simsek Papur, E. Dirkx, L. Wong, T. Sips, S. Wang, A. Strzelecka, M. Nabben, J. F. C. Glatz, D. Neumann, J. J. F. P. Luiken

**Affiliations:** 1 Department of Genetics and Cell Biology, Faculty of Health, Medicine and Life Sciences, Maastricht University Medical Center+, 6200 MD Maastricht, The Netherlands; 2 Department of Clinical Genetics, Faculty of Health, Medicine and Life Sciences, Maastricht University Medical Center+, 6200 MD Maastricht, The Netherlands; 3 Department of Pathology, Faculty of Health, Medicine and Life Sciences, Maastricht University Medical Center+, 6200 MD Maastricht, The Netherlands; 4 CARIM School for Cardiovascular Diseases, Maastricht, The Netherlands

**Keywords:** Diabetic cardiomyopathy, Glucose transport, GLUT4 translocation, Insulin resistance, Phosphatidylinositol-4-kinase-IIIβ

## Abstract

In the diabetic heart, long-chain fatty acid (LCFA) uptake is increased at the expense of glucose uptake. This metabolic shift ultimately leads to insulin resistance and a reduced cardiac function. Therefore, signaling kinases that mediate glucose uptake without simultaneously stimulating LCFA uptake could be considered attractive anti-diabetic targets. Phosphatidylinositol-4-kinase-IIIβ (PI4KIIIβ) is a lipid kinase downstream of protein kinase D1 (PKD1) that mediates Golgi-to-plasma membrane vesicular trafficking in HeLa-cells. In this study, we evaluated whether PI4KIIIβ is involved in myocellular GLUT4 translocation induced by contraction or oligomycin (an F_1_F_0_-ATP synthase inhibitor that activates contraction-like signaling). Pharmacological targeting, with compound MI14, or genetic silencing of PI4KIIIβ inhibited contraction/oligomycin-stimulated GLUT4 translocation and glucose uptake in cardiomyocytes but did not affect CD36 translocation nor LCFA uptake. Addition of the PI4KIIIβ enzymatic reaction product phosphatidylinositol-4-phosphate restored oligomycin-stimulated glucose uptake in the presence of MI14. PI4KIIIβ activation by PKD1 involves Ser294 phosphorylation and altered its localization with unchanged enzymatic activity. Adenoviral PI4KIIIβ overexpression stimulated glucose uptake, but did not activate hypertrophic signaling, indicating that unlike PKD1, PI4KIIIβ is selectively involved in GLUT4 translocation. Finally, PI4KIIIβ overexpression prevented insulin resistance and contractile dysfunction in lipid-overexposed cardiomyocytes. Together, our studies identify PI4KIIIβ as positive and selective regulator of GLUT4 translocation in response to contraction-like signaling, suggesting PI4KIIIβ as a promising target to rescue defective glucose uptake in diabetics.

**Electronic supplementary material:**

The online version of this article (10.1007/s00018-020-03669-7) contains supplementary material, which is available to authorized users.

## Introduction

In the healthy adult heart, there is an apparent balance between glucose and LCFA utilization: LCFA contribute to ~ 60% of the ATP production, while ~ 30% is derived from glucose. Deviation from this balance is associated with cardiac disease [1–4]. In hearts from rodent models with type 2 diabetes, uptake of LCFA is increased at the expense of glucose uptake. As a crucial step during the development of diabetic cardiomyopathy, CD36, the major cardiac LCFA transporter, permanently relocates to the sarcolemma, which increases LCFA uptake and subsequently myocellular lipid deposition, ultimately leading to inhibition of insulin signaling, as exemplified by decreased insulin-stimulated Akt-Ser473 phosphorylation [5]. Thereafter, GLUT4 translocation and myocellular glucose uptake are impaired [6]. Finally, lipid-induced insulin resistance leads to cardiac dysfunction [7–9]. The substrate disbalance in the lipid-overloaded diabetic heart may be normalized by stimulation of glucose uptake. Because insulin-induced GLUT4 translocation is defective in the type 2 diabetic heart [9, 10], signaling pathways involved in contraction-induced GLUT4 translocation may contain suitable targets to increase cardiac glucose utilization during insulin resistance [11].

Contraction-induced GLUT4 translocation requires the simultaneous input of two independent signaling pathways: the AMP-activated protein kinase (AMPK) pathway and the protein kinase D1 (PKD1) pathway [12–15]. AMPK, an energy-sensing kinase, is essential for contraction-induced GLUT4 translocation [12] and, therefore, has long time been regarded as an ideal target for antidiabetic treatment [11]. However, AMPK is also involved in contraction-induced CD36 translocation [8, 12]. Thus, AMPK activation unselectively stimulates both cardiac glucose and LCFA uptake, and, therefore, is not capable of rebalancing cardiac substrate preference in the insulin-resistant heart. PKD1 resides on the other signaling axis towards contraction-induced GLUT4 translocation. This kinase is the founding member of the PKC-related PKD family of Ser/Thr kinases [16]. PKD1 is activated via death-activated protein kinase (DAPK) during contraction, leading to GLUT4 translocation to the cell surface [13, 17]. Unlike AMPK, PKD1 is not involved in contraction-induced CD36 translocation [13], which provides PKD1 with the ability to shift cardiac substrate preference towards glucose. Indeed, PKD1 overexpression in cardiomyocytes in vitro or in the heart in vivo prevents the development of lipid-induced insulin resistance [18, 19]. Together, these findings provide proof-of-principle for the notion that selective up-regulation of glucose uptake is a possible anti-diabetic strategy. However, over-activation of cardiac PKD1 in absence of high-fat diet causes cardiac hypertrophy [20–22] through a mechanism involving phosphorylation of histone deacetylase-5 (HDAC5) and subsequent release of the transcription factor MEF2 to initiate hypertrophic programming [23]. Therefore, signaling modules downstream of PKD1 with involvement in contraction-induced GLUT4 translocation, but not triggering the undesired hypertrophic actions of PKD1, await their identification.

Phosphatidyl-inositol-4 kinase-IIIβ (PI4KIIIβ) is a downstream kinase of PKD1, which phosphorylates phosphatidylinositol species at the D4 position of the inositol ring, resulting in 4 possible products: PI[4]P, PI[3,4]P_2_, PI[4,5]P_2_, PI[3,4,5]P_3_) [24]. Each of these phosphatidylinositols has a specific action in vesicular trafficking by providing platforms for the recruitment of distinct sets of proteins in different vesicular compartments specialized in vesicle fission [25]. In secretory cells, PI4KIIIβ is present in the Golgi/endosomal network in physical interaction with PKD1, regulating vesicle-mediated cargo transport to the cell surface [24]. PKD1 not only associates with PI4KIIIβ but also directly phosphorylates the latter protein. Phosphorylation of Ser294 induces binding of 14-3-3 protein, which stabilizes PI4KIIIβ [26, 27] but is not necessary for the Golgi recruitment [24], resulting in the formation of various phosphatidylinositol species.

Based on the earlier findings described above, we hypothesize that PI4KIIIβ is involved in contraction-induced GLUT4 translocation in cardiomyocytes and hence could serve as anti-diabetic target. In this study, we used electric pacing and oligomycin as contraction-like stimuli [12]. We first explored whether PI4KIIIβ is involved in contraction-induced GLUT4 translocation. Second, we investigated the effect of adenoviral overexpression of PI4KIIIβ wild type (WT), Ser294Ala phosphorylation site mutant (S294A) and Asp656Ala kinase-deficient mutant (D656A) on glucose uptake in cardiomyocytes. Third, we studied the activation mechanism (e.g., phosphorylation, activity, and/or localization) of PI4KIIIβ involved in contraction-induced GLUT4 translocation in cardiomyocytes. Finally, we investigated whether adenoviral overexpression of PI4KIIIβ WT, or S294A and D656A mutant, can preserve insulin sensitivity in insulin-resistant cardiomyocytes. The combined studies identify PI4KIIIβ as a positive selective regulator of contraction-induced GLUT4 translocation and subsequent glucose uptake in cardiomyocytes.

## Materials and methods

### Materials

[1-^14^C]Palmitic acid and [1-^3^H]deoxyglucose were obtained from GE Healthcare. Laminin and insulin were purchased from Sigma-Aldrich. Bovine serum albumin (BSA) (fraction V), dependent on the application, was derived from MP Biomedicals (for cell isolation and incubation purposes), or from Sigma-Aldrich (other purposes). Collagenase type II was from Worthington. PI4P, PI[4,5]P_2_, unlabeled shuttle PIP carrier 3 and PI4-Kinase activity assay kit were obtained from Echelon Biosciences and used based on their protocols. Antibodies directed against insulin-regulated aminopeptidase (IRAP), PKD1/PKCμ, phospho-ERK (Thr202/Tyr204), phospho-PKD1/PKC-μ (Ser916), phospho-Akt (Ser473) and phospho-troponin-I (Ser23/24) were obtained from Cell Signaling Technology. Phospho-ACC (Ser79) antibody was obtained from Upstate. Phospho-HDAC5 (Ser498) was from Abcam. PI4KIIIβ antibody and mouse CD36 antibody were obtained from Millipore. Phospho-PI4KIIIβ (Ser294) antibody was kindly provided by Dr. A. Hausser (University of Stuttgart, Stuttgart, Germany). Anti-CD36 antibody MO25 was kindly provided by Dr. N.N. Tandon (Otsuka Maryland Research Institute Rockville, Rockville, USA). Antibodies against Caveolin3 (Cav3) and GAPDH were obtained from BD transduction laboratories. Pan 14-3-3 antibody was from Santa Cruz Biotechnology. Claycomb medium, fetal bovine serum (FBS), norepinephrine, oligomycin, okadaic acid, phorbol-12-myristate-13-acetate, staurosporine were purchased from Sigma-Aldrich. Dulbecco’s modified Eagles medium (DMEM), l-glutamine, non-essential amino acids (NEAA), penicillin and streptomycin (PenStrep), Lipofectamine-2000 and phosphate-buffered saline (PBS) were from Thermo Fisher Scientific. MI14 was obtained from TOCRIS. Small-interfering RNAs (siRNA) against PI4KIIIβ (GCCAUAUAAGAUUCUUGUGtt) was from Ambion (AM16708, lot#: ASO225QB). The plasmid for GLUT4-myc was kindly provided by Dr. J. Eckel (German Diabetes Center, Düsseldorf, Germany). ADP-Glo™ kinase assay was purchased from Promega.

### Adenovirus amplification

Control adenovirus containing enhanced green fluorescent protein (GFP) (Ad GFP) and a recombinant adenovirus encoding full-length wild-type mouse PKD1 and EGFP (Ad PKD1) were obtained and amplified as previously described [18]. Plasmid constructs encoding full-length human PI4KIIIβ (wild type, S294A, D656A) were kindly provided by Dr. A. Hausser (University of Stuttgart, Stuttgart, Germany). Adenovirus encoding full-length human PI4KIIIβ (wild type, S294A, D656A) (Ad PI4KIIIβ, Ad PI4KIIIβ S294A, Ad PI4KIIIβ D656A) was generated from the plasmid constructs above with AdEasy Adenoviral Vector System (Agilent Technologies) and amplified by the method as previously described [18]. Optimal multiplicity of infection (MOI) was determined by western blotting for PKD1 and PI4KIIIβ.

### Experimental animals, isolation, and culturing of primary adult rat cardiomyocytes (aRCMs)

Male Lewis rats, 250–340 g, were purchased from Charles River laboratories, maintained at the Experimental Animal Facility of Maastricht University and used for isolation and subsequent culturing of cardiomyocytes. All animal procedures were approved with UM-project license number: PV-2016-004 by the Experimental Animal Committee of Maastricht University, NL.

aRCMs were isolated using a Langendorff perfusion system primarily yielding ventricular myocytes [28]. After isolation of aRCMs, 100,000 cells were seeded per well in laminin coated plates and after 90 min adhesion medium was replaced with either low palmitate medium (LP, 20 μM palmitate, palmitate:BSA 0.3:1), or high palmitate medium (HP, 200 μM, palmitate:BSA 3:1). Cells were cultured according to the method described by Volz et al. [29] in modified serum-free medium M199 containing carnitine, creatine and taurine [29]. Culturing adult cardiac myocytes does not affect their insulin responsiveness [30]. Cells were maintained in an incubator (37 °C, 5% CO2) and were used for experiments 48 h after seeding and transduction. aRCMs were transduced with Ad GFP, Ad PKD1, Ad PI4KIIIβ, Ad PI4KIIIβ S294A, or Ad PI4KIIIβ D656A 90 min after seeding aRCMs. aRCMs were cultured for 48 h because of optimal combination of MOI and transduction time.

### Cell culture of HL-1 cardiomyocytes and transfection

The atrial HL-1 cell line shows similar metabolic responses compared to primary cardiomyocytes [31]. Compared to aRCM, HL-1 cells are much easier to transfect with conventional transfection methods, such as with lipofectamine. Moreover, HL-1 cells are advantageous in subcellular fractionation studies, because of the extensive contractile apparatus present in aRCM that impairs the fractionation process. HL-1 cells were cultured and maintained in growth medium (Claycomb medium supplemented with 10% FBS, 0,1 mM norepinephrine, 2 mM l-glutamine, 100 U/ml penicillin and 100 µg/ml streptomycin) at 37 °C in a fully humidified atmosphere of 5% CO_2_.

HL-1 cells were seeded at a density of 600,000 cells per 9.5 cm^2^ (6 well) or 150,000 cells per 1.9 cm^2^ (24 well). After 24 h medium was replaced with transfection medium (Claycomb medium supplemented with 10% FBS and 1% l-glutamine). Cells were transfected with 300 pmol (6 well), 25 pmol, 50 pmol or 75 pmol (24 well) non-coding (n.c.) or PI4KIIIβ siRNA with Lipofectamine 2000 and subsequently incubated at 37 °C for 6 h. Transfection medium was replaced with growth medium after incubation and cells were able to grow for 48 h. For detection of GLUT4 in the translocation assay, a GLUT4 variant carrying a myc-tag (1.2 µg) on its first extracellular epitope was co-transfected together with the siRNA.

### Human-induced pluripotent stem cell (hiPSC) maintenance and differentiation into cardiomyocytes (hiPSC-CMs)

hiPSC were cultured and differentiated into cardiomyocytes as previously described [32].

### CD36 and GLUT4 translocation assay

GLUT4-myc transfected HL-1 cells (600,000 cells per 9.5 cm^2^) were incubated in depletion medium (DMEM low glucose, supplemented with 2 mM l-glutamine, 100 µM NEAA) for 16 h before they were electrically paced for 30 min (40 V, 2 Hz 15 ms), using the C-Pace Stimulator and 6-well C-Dish Culture Dish Electrodes from IonOptix.

After that, cells were fixed in 4% formaldehyde for 10 min at room temperature and blocked for 1 h with PBS containing 1% non-fat-dry-milk (nfdm) and 0.5% Tween20. Subsequently, cells were incubated with an anti-CD36 (1:2000 in blocking buffer) and anti-myc antibody (1:1000 in blocking buffer) for 30 min at room temperature. Next, cells were incubated with an HRP-linked secondary antibody (1:4000 in blocking buffer) for 30 min at room temperature. After extensive washing with blocking buffer and PBS, an ortho-phenylenediamine-H_2_O_2_ (OPD) solution was added as a substrate for the bound HRP. The reaction was carried out at room temperature and stopped after 30 min by addition of 1 M H_2_SO_4_. Color development, representative for the amount of CD36 or GLUT4 present at the plasma membrane, was quantified by measurement of the absorbance at 490 nm. The background signal of control (incubation without primary antibody) was subtracted from the raw data.

### Measurement of substrate uptake

Uptake of [1-^14^C]palmitate (in complex with BSA) and [1-^3^H]deoxyglucose into suspensions of cardiomyocytes was measured as previously described [33]. Uptake of these substrates into HL-1 cardiomyocytes and into cultured aRCMs was measured as previously described for cardiomyocytes in culture [31, 34].

### Sarcolemmal CD36 and IRAP content of aRCMs

Surface-protein biotinylation was measured as previously described [35]. Directly after isolation, cells were plated in laminin-coated 6-well plates. After 90 min, cells were preincubated with or without 2.5 µM MI14 for 7 min. After that, 5 µM oligomycin was added, or not, to the cells to incubate for 15 min. Then, cells were washed twice briefly with ice-cold PBS, followed by 1 h incubation in sulfo-NHS-LC-biotin in PBS (1 mg/ml) on ice. Cells were treated with ice-cold glycine in PBS (100 mM) to quench and remove excess biotin. After a brief wash with ice-cold PBS, cells were lysed by scraping in 200 μl lysis-buffer containing 1% Triton-X100. The lysates were incubated on ice for 10 min, and centrifuged for 10 min at 16.1 × 1000*g* at 4 °C. A 20 μl supernatant sample was used as total lysate sample for western blotting, and 180 μl was inverted gently for 2 h at 4 °C in the presence of 50 μl immobilized streptavidin to allow the streptavidin to bind biotinylated proteins. Samples were centrifuged for 2 min at 16.1 × 1000*g*, and streptavidin beads were washed twice with lysis-buffer containing 1% Triton-X100. Biotinylated proteins were eluted by incubation of the streptavidin beads for 5 min at 95 °C in 40 μl Laemmli sample buffer. Samples (30 μl) were analyzed by SDS-PAGE, followed by western blotting for the detection of IRAP (1: 1000 in 5% BSA in TBST; Cell Signaling Technology) and CD36 (1: 20,000 in 7.5% BSA in TBST; A gift from Dr. N.N. Tandon). IRAP is an abundant cargo protein associated with GLUT4 vesicles that translocates in response to hormonal/metabolic stimuli in a manner identical to GLUT4 [36]. Therefore, IRAP detection following the biotinylation assay is a suitable readout for GLUT4 translocation [37, 38]. Biotinylated proteins were quantified by densitometry of unadjusted western blots from at least three independent experiments using Quantity One 4.6.3 Basic software (BioRad; available on the Internet at http://www.bio-rad.com).

### Cell lysis and western blotting

Cell lysis was performed as described earlier [31]. Protein concentrations were determined by BCA protein assay (Thermo Scientific). Equal amounts of cell lysates were subjected to SDS/PAGE (200 V for 45 min) and electrotransferred to nitrocellulose membranes (100 V for 75 min). Membranes were blocked in a 5% non-fat dry milk solution for 1 h and were then probed overnight at 4 °C with the primary antibodies against p-ACC (Ser79), PKD1, p-PKD1(Ser916), p-ERK(Thr202/Tyr204), p-AKT(Ser473), GAPDH, PI4KIIIβ, p-PI4KIIIβ(Ser294), p-HDAC5(Ser498), IRAP, CD36, 14-3-3 and Cav3. Thereafter, blots were probed with the secondary antibody anti-rabbit IgG HRP linked (Cell Signaling Technology). SwaRpo P0399 (DakoCytomation) was used as a secondary antibody for p-ACC(ser79). Rampo P0161 (DakoCytomation) was used as a secondary antibody for CD36 (MO25) and Cav3. Anti-mouse IgG HRP linked (Cell Signaling Technology) was used as a secondary antibody for 14-3-3. DarPo 711-035-152 (Jackson Immuno Laboratories Inc) was used as a secondary antibody for p-HDAC5. Blots were detected using enhanced chemiluminescence (Sigma-Aldrich) and quantified by densitometric analyses (Quantity one, Biorad). To ensure equal loading of proteins, Cav3 or GAPDH was used as loading control.

### PI4KIIIβ immunoprecipitation and PI4KIIIβ activity assay in cardiomyocytes

Isolated aRCMs were incubated for 15 min at 37 °C with or without 5 μM oligomycin. Thereafter the cells were pelleted and resuspended in ice-cold lysis buffer (50 mM Tris HCl (pH 7.4), 2 mM EDTA, 2 mM EGTA, 2 mM dithiothreitol, 4% (vol/vol) complete protease inhibitor cocktail, 5% PhosSTOP phosphatase inhibitor cocktail and 1% (w/v) Triton X-100). Briefly, cell suspensions were centrifuged at 16.1 × 1000*g* for 30 min at 4 °C. Supernatant fractions (500 μl) were immunoprecipitated with PI4KIIIβ antibody (1:100) overnight at 4 °C on a rotating wheel. Thereafter, 100 μl of Protein A Agarose bead (Millipore) suspension was added and incubations were continued for 2 h at 4 °C on a rotating wheel. The beads were collected (7000 rpm × 1 min) and washed twice with lysis buffer, twice with kinase assay buffer (30 mM Tris, pH 7.4, 15 mM MgCl_2_). Thereafter, the beads were used for PI4KIIIβ activity assay by both ADP Glo™ kinase assay kit (Promega) and PI4-Kinase activity assay kit (Echelon) as described by the online protocols. PI4KIIIβ from ProQinase was used as positive control for ADP Glo™ kinase assay kit.

### Subcellular fractionation

HL-1 cardiomyocytes were fractionated into membrane and cytosolic fractions, as previously described [39], for further analysis by western blotting.

### Immunofluorescence and confocal imaging

HL-1 cardiomyocytes were cultured on gelatin/fibronectin-coated coverslips in 24-well plate. Prior to immunofluorescence, cells were incubated in serum-free depletion medium for 16 h. Next day, cells were incubated with or without 1 µM oligomycin for 30 min. After that, cells were washed twice with PBS and fixed with 4% formaldehyde afterwards for 15 min at room temperature. Subsequently, cells were rinsed twice in PBS and permeabilised by 1% Triton X-100 in PBS for 15 min at room temperature, followed by 30 min of incubation with 1% BSA in PBST. Then, cells were incubated with anti-PI4KIIIβ antibody (BD biosciences, 1:12.5) and anti-v-ATPase a2 (Abcam; 1:12.5) in 1% BSA in PBST for 1 h at room temperature, followed by Alexa Fluor 488 goat anti-rabbit secondary antibody (Thermo Fisher, 1:500) and Alexa Fluor 647 donkey anti-mouse secondary antibody (Thermo Fisher, 1:500) in 1% BSA in PBST for 1 h at room temperature in darkness. Nuclei were stained with Hoechst 33342 (Thermo Fisher, 1:1000) for 2 min. Coverslips were washed and mounted with antifade mounting medium (Vector Laboratories). Cells on coverslips were imaged using a Leica TCS SPE confocal laser scanning microscope (Leica Microsystems GmbH) equipped with diode lasers of 405, 488, 532 and 635 nm, using oil immersion objectives (63 ×, numerical aperture = 1.4). Optical sections were recorded with three scans for each image. ImageJ software was used to analyse the images. Image brightness and contrast was adjusted to the same settings where needed.

### RNA isolation and RT-PCR

Total RNA was isolated using Trisure (Bioline) and 1 µg cDNA was synthesized using the SensiFAST cDNA synthesis kit (Bioline). Relative gene expression was determined by RT-PCR using Sensimix SYBR & Fluorescence kit (Bioline) and the Roche LightCycler 480 II real-time PCR System. BNP gene was analyzed (5′ → 3′): forward: AGGAGAGACTTCGAAATTCCAAGA; reverse: CTAAAACAACCTCAGCCGTCA. The ∆∆ CT method was used for quantification and samples were normalized against the housekeeping gene GAPDH (forward: GGGTG TGAACCACGAGAAAT; reverse: ACTGTGGTCATGAGCCCTC) and Cyclophilin A (forward: TTCCTCCTT TCACAGAATTATTCCA; reverse: CCGCCAGTGCCATTATGG).

### Statistics

All data are presented as mean ± SEM. Statistical analysis was performed using two-sided Student’s *t* test, or when possible we applied paired testing. *P* values of less than 0.05 were considered statistically significant.

## Results

**Fig. 1:**
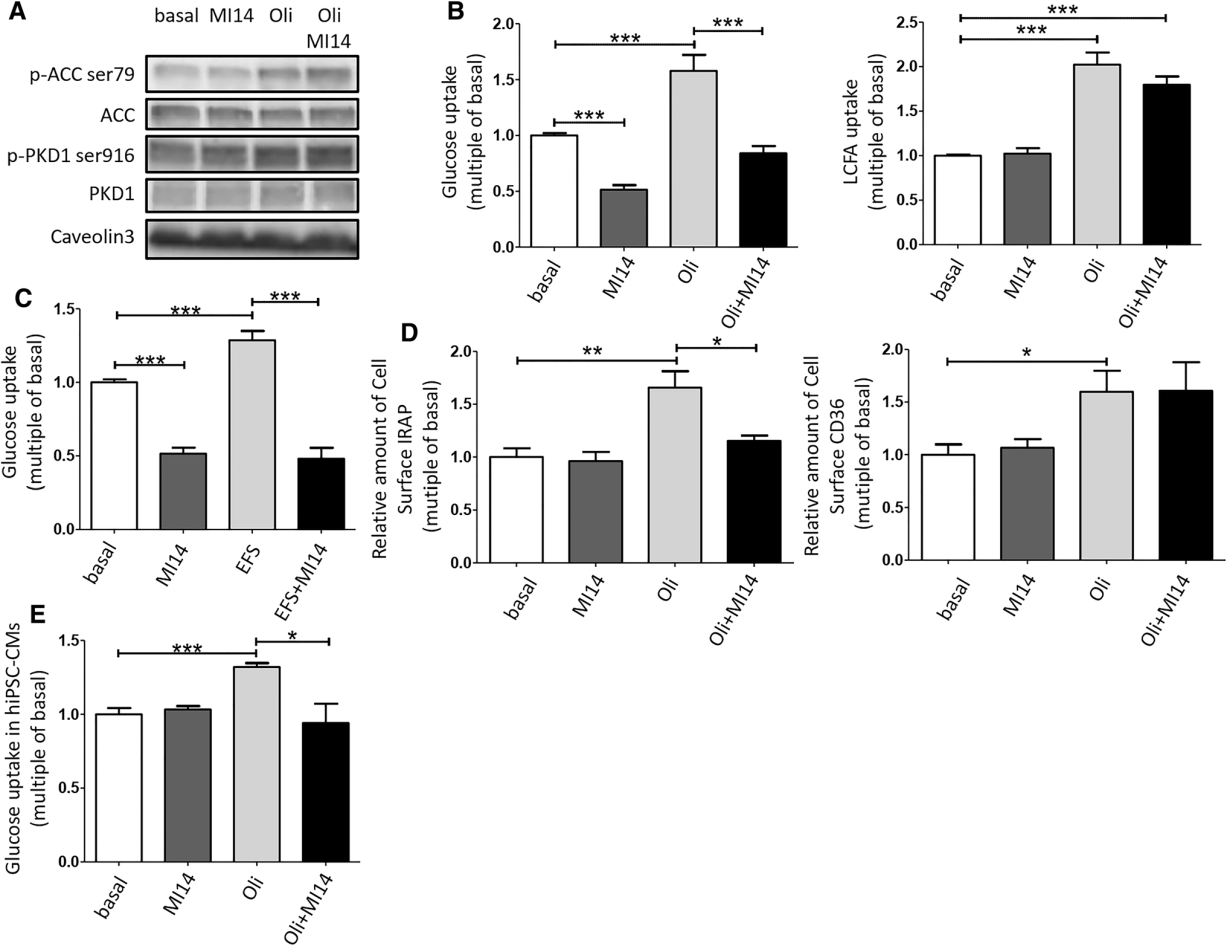
PI4KIIIβ inhibition blocks contraction-induced glucose uptake but not fatty acid uptake. Oligomycin (Oli)/electrostimulation-induced AMPK/PKD1 pathway, glucose/LCFA uptake, and GLUT4/CD36 translocation was determined in adult rat cardiomyocytes (aRCMs) in absence and presence of the pharmacological PI4KIIIβ inhibitor MI14. **a**, **b** Freshly isolated aRCMs were pretreated with or without 2.5 µM MI14 for 7 min followed by a treatment with or without 5 µM Oli for 15 min. **a** A representative western blot of ACC ser79 phosphorylation, total ACC, PKD1 ser916 phosphorylation and total PKD1. Caveolin3 was the loading control (*n* = 3). **b** Glucose and LCFA uptake (*n* = 5). **c** Glucose uptake. Freshly isolated aRCMs were pretreated with or without 2.5 µM MI14 for 7 min followed by a treatment with or without 4 Hz electric field stimulation (EFS) for 5 min (*n* = 4). **d** Relative amount of cell surface insulin-regulated aminopeptidase (IRAP, which reflects GLUT4) (left, *n* = 7)/CD36 (right, *n* = 3). Isolated aRCMs were seeded and pretreated with or without 2.5 µM MI14 for 7 min followed by a treatment with or without 5 µM Oli for 15 min. **e** Glucose uptake in human iPSC-derived cardiomyocytes (hiPSC-CMs). hiPSC-CMs were pretreated with or without 2.5 µM MI14 for 7 min followed by a treatment with or without 1 µM Oli for 15 min (*n* = 3). Graphs depict means ± SEM. *P < 0.05, **P < 0.005, ***P < 0.001. Data were normalized to basal

**Pharmacological and genetic inhibition of PI4KIIIβ decreases contraction-induced GLUT4 translocation and glucose uptake, but not CD36 translocation and LCFA uptake**. To investigate whether PI4KIIIβ is involved in contraction-induced GLUT4 translocation and glucose uptake, freshly isolated aRCMs were preincubated with the specific PI4KIIIβ inhibitor MI14 [40] followed by stimulation with oligomycin. Oligomycin was used as contraction-mimetic stimulus because the metabolic and signaling signature upon treatment with this compound is very similar to that of electric field-induced contraction, including decrease in energy status and activation of the signaling kinases AMPK and PKD1 [12, 13, 15]. Moreover, both contraction and oligomycin have been shown to stimulate myocellular glucose and LCFA uptake in a non-additive manner, indicating that the same mechanisms are involved [12]. First, we assessed the effects of MI14 on contraction-like signaling in aRCMs. Oligomycin treatment resulted in increased phosphorylation of both ACC (at Ser79, a readout for AMPK activation) and of PKD1 (at Ser916, a readout for PKD1 activation) (Fig. 1a), in agreement with earlier work [13]. Phosphorylation of both proteins was not altered by MI14, neither in absence nor in presence of oligomycin indicating that its inhibitory action on PI4KIIIβ is not due to a blockade of direct upstream signaling (Fig. 1a). Subsequently, MI14 was used to study the involvement of PI4KIIIβ in contraction/oligomycin-induced myocellular substrate transport and transporters. Insulin-responsive aminopeptidase (IRAP) has been known to be associated with GLUT4 vesicles, and its surface localization is a suitable readout of GLUT4 translocation [41]. Oligomycin treatment stimulated myocellular glucose uptake and GLUT4 translocation, as well as LCFA uptake and CD36 translocation, by > 1.5-fold (Fig. 1b, d), in agreement with previous observations [13, 18]. MI14 inhibited oligomycin-stimulated glucose uptake and GLUT4 translocation, whereas LCFA uptake and CD36 translocation were not affected (Fig. 1b, d). Furthermore, 4 Hz electrostimulation increased myocellular glucose uptake (1.3-fold), which was abolished by MI14 pretreatment (Fig. 1c).

To establish whether PI4KIIIβ is also involved in contraction-induced myocellular glucose uptake in the human setting, we used human-induced pluripotent stem cells transdifferentiated to cardiomyocytes (hiPSC-CM). The cardiomyocyte phenotype was established as recently described [32]. Similarly, as observed for rodent cardiomyocytes, MI14 treatment inhibited oligomycin-stimulated uptake of glucose in hiPSC-CM (Fig. 1e).

**Fig. 2:**
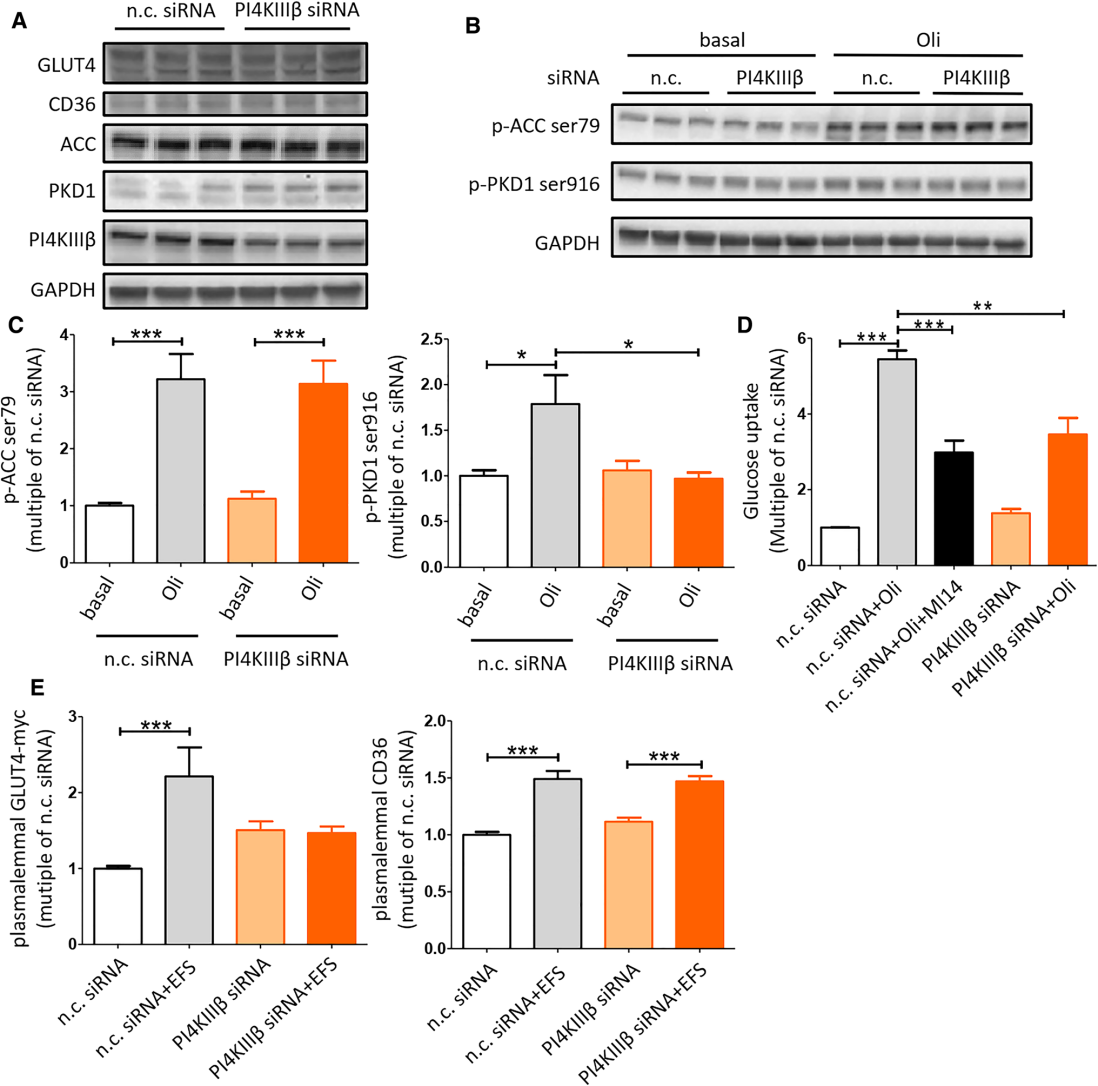
Genetic silencing of PI4KIIIβ inhibits contraction-induced glucose uptake and translocation. Oligomycin (Oli)/electrostimulation-induced AMPK/PKD1 pathway, glucose uptake, GLUT4 and CD36 translocation was determined after siRNA-mediated silencing of PI4KIIIβ in HL-1 cells. Therefore, HL-1 cardiomyocytes were cultured for 48 h after transfection with non-coding (n.c.) siRNA or PI4KIIIβ siRNA. **a** A representative western blot of GLUT4, CD36, ACC, PKD1 and PI4KIIIβ (*n* = 3). GAPDH was the loading control. **b** A representative western blot of ACC Ser79 phosphorylation and PKD1 Ser916 phosphorylation (*n* = 3). GAPDH was the loading control. **c** On the left: quantification of the level of phosphorylation at Ser79 site of ACC. On the right: quantification of the level of phosphorylation at Ser916 site of PKD1 (*n* = 3). **d** Glucose uptake (*n* = 3). HL-1 cells were pretreated with or without 2.5 µM MI14 for 15 min followed by a treatment with or without 1 µM Oli for 30 min. **e** GLUT4 and CD36 translocation (*n* = 3). HL-1 cells were treated with or without 2 Hz electric field stimulation (EFS) for 30 min. Graphs depict means ± SEM. **P < 0.005, ***P < 0.001. Data were normalized to n.c. siRNA

To obtain genetic evidence for the involvement of PI4KIIIβ in contraction-induced GLUT4 translocation and subsequent glucose uptake, PI4KIIIβ was silenced by siRNA in HL-1 cardiomyocytes. Knockdown of PI4KIIIβ was verified by western blotting: a 60% decrease in protein expression by 25 pmol PI4KIIIβ siRNA, with no further decrease at higher siRNA concentrations (Fig. S2). At 25 pmol, PI4KIIIβ siRNA had no effect on GLUT4, CD36 and ACC content, but increased PKD1 content (Fig. 2a). In addition, reduced PI4KIIIβ expression did not affect oligomycin-stimulated ACC-Ser79 phosphorylation but impaired oligomycin-stimulated PKD1-Ser916 phosphorylation (Fig. 2b, c). Hence, PI4KIIIβ silencing can be used to assess the metabolic effects of this lipid kinase independently of AMPK, but not of PKD1. PKD1 downregulation by PI4KIIIβ silencing is surprising given that PI4KIIIβ is downstream of PKD1, thus raising the possibility of a feedback mechanism. With respect to metabolism, PI4KIIIβ siRNA inhibited oligomycin-stimulated glucose uptake to a similar extent as MI14 (Fig. 2d). In agreement with our previous observations [13], 2 Hz electrostimulation increased cell surface content of both GLUT4 and CD36 by > 1.5-fold (Fig. 2e). PI4KIIIβ siRNA diminished contraction-induced GLUT4 translocation but did not alter contraction-induced CD36 translocation (Fig. 2e). Together, these results provide strong evidence for a crucial and specific role of PI4KIIIβ in contraction-induced GLUT4 translocation and myocellular glucose uptake.

**Fig. 3:**
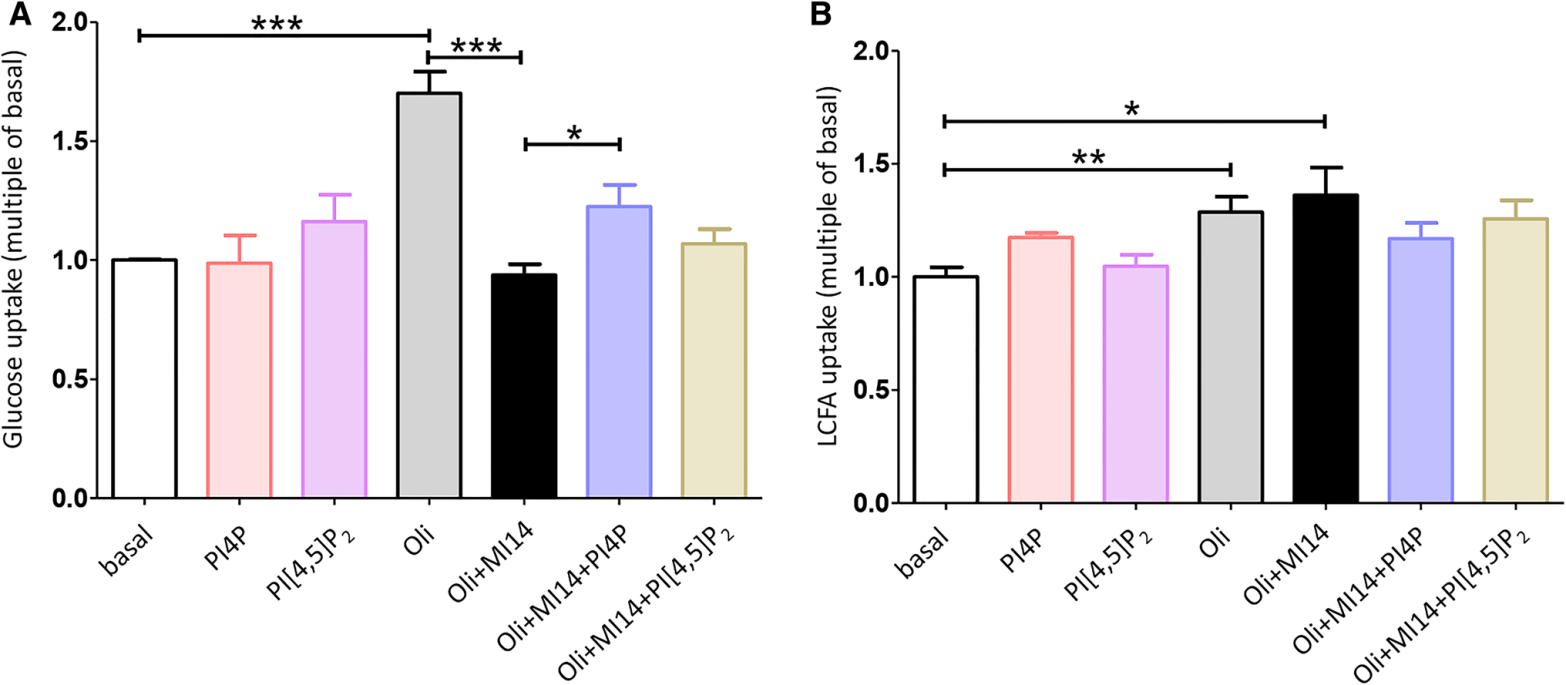
Supplementation with the PI4KIIIβ enzyme product PI4P triggers glucose uptake. Oligomycin (Oli)-stimulated glucose and LCFA uptake in adult rat cardiomyocytes (aRCMs) was inhibited with MI14 and then measured with or without delivery of exogenous PI4P and PI[4,5]P_2_. Freshly isolated aRCMs were pretreated with or without 2.5 µM MI14 for 7 min followed by a treatment with or without 5 µM Oli in addition with either 20 µM PI4P or PI[4,5]P_2_ for 15 min. **a** Glucose uptake (*n* = 4). **b** LCFA uptake (*n* = 4). Graphs depict means ± SEM. *P < 0.05, ***P < 0.001. Data were normalized to basal

**Addition of PI4P, not PI[4,5]P**_**2**_**, restores contraction-induced glucose uptake in the presence of MI14 in rat cardiomyocytes.** To investigate whether PI4KIIIβ regulates contraction-induced glucose uptake in cardiomyocytes via its products, aRCMs were preincubated with or without MI14 followed by treatment with or without oligomycin, together with one of two PI4KIIIβ products: PI4P di-C16 or PI[4,5]P_2_ di-C16. These PIPs were added at a concentration of 20 µM in complex with histone, which is an established lipid delivery method [42, 43]. Addition of PI4P or PI[4,5]P_2_ had no effect on basal glucose uptake. In contrast, addition of PI4P partly restored oligomycin-stimulated glucose uptake after blockade by MI14, whereas no significant effect of PI[4,5]P_2_ was observed (Fig. 3a). Both PIPs did not alter LCFA uptake, neither by themselves nor upon oligomycin stimulation in combination with MI14-blockade (Fig. 3b). Please note that the oligomycin stimulation of LCFA uptake in aRCMs is lower than in Fig. 1b, yet it is still significant. Taken together, the data suggest that PI4KIIIβ mediates contraction-induced glucose uptake, at least in part, through formation of its enzyme product PI4P.

**Fig. 4:**
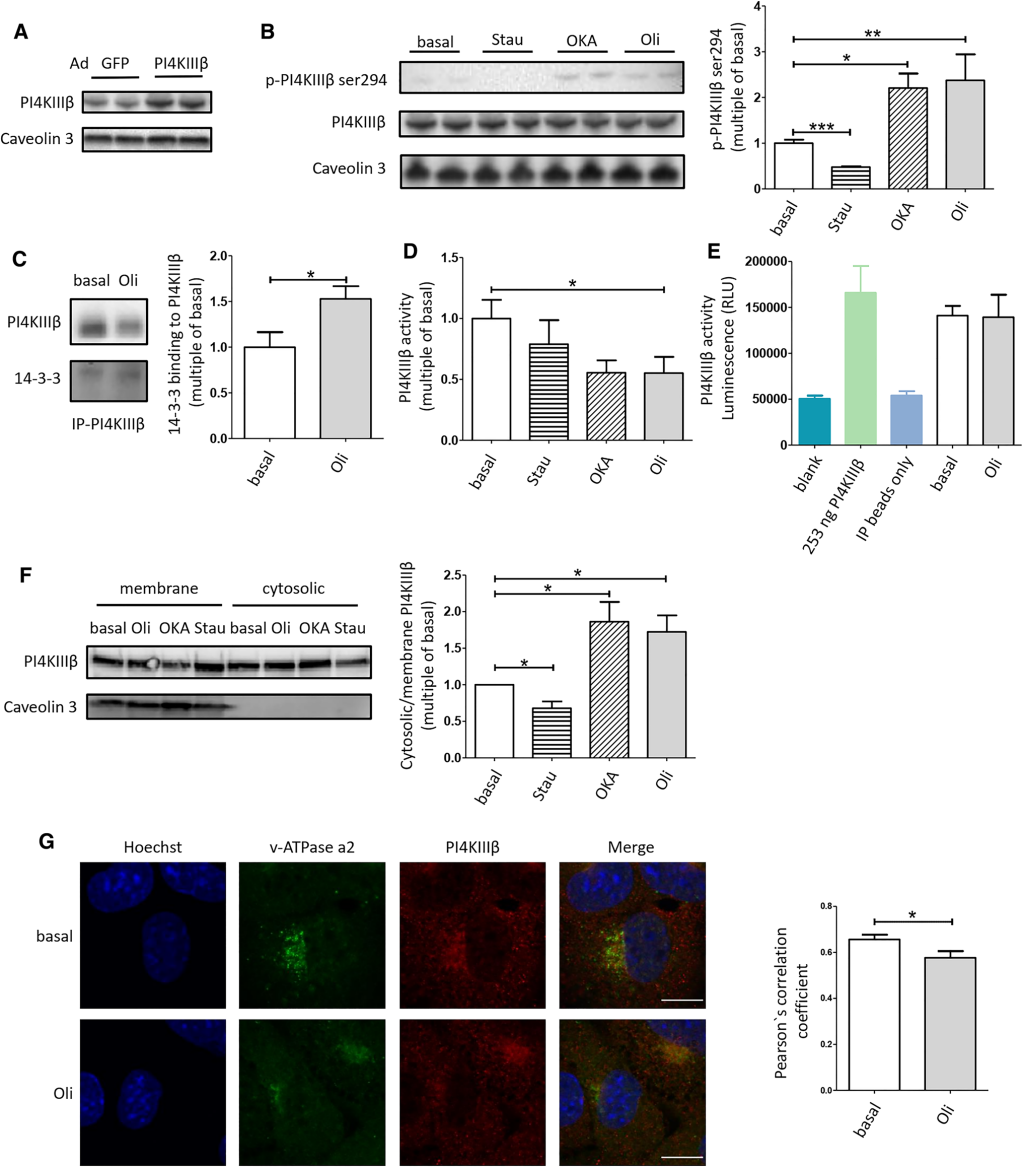
Contraction-mimetic signaling triggers PI4KIIIβ phosphorylation and localization changes. Phosphorylation at Ser294 site, binding to 14-3-3, activity, and localization of PI4KIIIβ was measured in response to oligomycin (Oli) treatment. **a**, **b** HL-1 cells were cultured for 48 h after transduction with adenoviral PI4KIIIβ wild type, followed by an incubation with 1 µM staurosporine (Stau), 1 µM okadaic acid (OKA), or 1 µM Oli for 30 min. Cells were lysed and western blotting was performed with antibodies against p-PI4KIIIβ Ser294, PI4KIIIβ. **a** A representative western blot of PI4KIIIβ protein overexpression in HL-1 cells. **b** One the left: a representative western blot of phosphorylation at Ser294 site of PI4KIIIβ and total PI4KIIIβ after adenoviral overexpression. On the right: quantification of the level of phosphorylation at Ser294 site of PI4KIIIβ (*n* = 3). In all panels, Caveolin3 protein content serves as loading control. **c** On the left: a representative western blot of 14-3-3 and endogenous PI4KIIIβ after immunoprecipitation with PI4KIIIβ antibody. On the right: quantification of the binding of 14-3-3 to PI4KIIIβ. Adult rat cardiomyocytes (aRCMs) were incubated with or without 5 µM Oli. Binding of 14-3-3 to endogenous PI4KIIIβ was measured by western blotting and quantified by densitometric analyses (Quantity one, Biorad) (*n* = 3). **d**, **e** Endogenous PI4KIIIβ activity. **d** aRCMs were incubated with either 5 µM Oli, 1 µM OKA or 1 µM Stau. PI4KIIIβ acitivity assay kit from Echelon Biosciences was used to measure PI4KIIIβ activity (*n* = 4). **e** HL-1 cells were incubated with or without 1 µM Oli. ADP-Glo™ kinase assay from Promega was used to measure PI4KIIIβ activity. 253 ng PI4KIIIβ from was used as positive control, and IP beads were used as negative control (*n* = 4). **f** Localization of endogenous PI4KIIIβ. On the left: a representative western blot of membrane and cytosolic PI4KIIIβ. Caveolin3 was used as a positive control for membrane fraction. On the right: quantification of the cytosolic/membrane PI4KIIIβ ratio. HL-1 cells were incubated with 1 µM Stau, 1 µM OKA, or 1 µM Oli for 30 min. Contents of PI4KIIIβ were assessed by western blotting of the cytosolic fraction and membrane fraction. Cytosolic/membrane ratio was used for the quantification (*n* = 4). **g** Colocalization of PI4KIIIβ and v-ATPase a2. On the left: immunofluorescence of PI4KIIIβ and v-ATPase a2. Indicated HL-1 cells were incubated with or without 1 µM Oli for 30 min and fixed. PI4KIIIβ (shown in red) and v-ATPase a2 (shown in green, as a marker for endosomes membrane) were immune stained using the respective protein-specific antibodies detected by Alexa Fluor 647 donkey anti-mouse secondary antibody and Alexa Fluor 488 goat anti-rabbit secondary antibody, respectively. Cells were stained with Hoechst 33342 for visualization of nuclei (shown in blue). Fluorescent staining was assessed by confocal imaging (there is no cross talk between the fluorescent channel for Alexa Fluor 647 and the fluorescent channel for Alexa Fluor 488). Data are representative of three experiments. Images have been adjusted for brightness and contrast for presentation. Scale bars correspond to 10 µm. On the right: quantification of Pearson`s correlation coefficient. The bar graph shows a comparison of basal condition versus Oli treatment (*n* = 3). Graphs depict means ± SEM. *P < 0.05. Data were normalized to basal

**Oligomycin treatment enhanced phosphorylation of PI4KIIIβ at Ser294 and binding between PI4KIIIβ and 14-3-3, but not kinase activity of PI4KIIIβ in cardiomyocytes.** To examine the activation mechanism of PI4KIIIβ in contraction-stimulated glucose uptake, we first tested the effect of 30 min oligomycin treatment on phosphorylation of PI4KIIIβ at Ser294. Phosphorylation at this site has been found to be induced by PKD1 overexpression and leads to increased kinase activity [24]. First, we assessed phosphorylation of endogenous PI4KIIIβ in HL-1 cells. As positive control, we used okadaic acid (a serine/threonine phosphatase PP2A inhibitor; 30 min treatment), which has been previously shown to induce PI4KIIIβ-Ser294 phosphorylation and activation [26], and as negative control staurosporine (a general protein kinase inhibitor) was applied. These first experiments did not yield any detectable signals upon western blotting under neither condition. We then sought to overexpress PI4KIIIβ in HL-1 cells using an adenoviral vector, which elevated the protein contents of PI4KIIIβ 48 h after transduction (Fig. 4a). In transduced cells, we found that oligomycin treatment stimulated PI4KIIIβ-Ser294 phosphorylation compared to basal. This increase in phosphorylation (2.4-fold) is of the same magnitude as observed for okadaic acid-stimulated cells (2.2-fold) (Fig. 4b).

Additionally, because it has been reported that PI4KIIIβ when phosphorylated at Ser294 binds to the adaptor protein 14-3-3 and improves kinase activity of PI4KIIIβ by stabilizing it [26], we assessed the effect of contraction-like signaling on the interaction between PI4KIIIβ and 14-3-3 in aRCMs. For this, the cells were incubated without or with oligomycin, followed by immunoprecipitation against PI4KIIIβ. Binding of 14-3-3 to PI4KIIIβ was assessed by Western blotting detection of 14-3-3 in the immunoprecipitate. Indeed, oligomycin treatment increased the interaction between PI4KIIIβ and 14-3-3 (Fig. 4c). This indicates that phosphorylation of PI4KIIIβ at Ser294 induced by contraction-like signaling resulted in binding to 14-3-3.

Next, we tested the effect of contraction-like signaling on PI4KIIIβ kinase activity in aRCMs and in HL-1. Cells were incubated without or with oligomycin followed by immunoprecipitation of PI4KIIIβ. The obtained immunoprecipitate was subjected to two different methods either measuring ADP production indicative of kinase activity (Fig. 4d) or detecting the formation of PI4P from PI (Fig. 4e). The adequacy of the second method was verified by the appropriate detection of 253 ng PI4KIIIβ. In both methods, oligomycin did not stimulate PI4KIIIβ activity (Fig. 4d, e). Also, okadaic acid failed to do so (Fig. 4d). Hence, it appears that stimulation of contraction-like signaling in cardiomyocytes increases Ser294 phosphorylation, but does not directly regulate the enzymatic activity of PI4KIIIβ.

Another possible mechanism of stimulation of PI4KIIIβ by contraction-like signaling may be the facilitation of access to its substrate (being PI in case of PI4P formation), which would imply altered localization. For this, HL-1 cardiomyocytes were fractionated directly after stimulation with oligomycin or okadaic acid. Remarkably, both okadaic acid and oligomycin treatment induced a PI4KIIIβ redistribution from the membrane fraction to the cytoplasm (Fig. 4f). We also assessed the degree of colocalization between PI4KIIIβ and the membrane localized V_0_-subcomplex of v-ATPase (marker of endosomes) by immunofluorescence microscopy. This colocalization decreased upon oligomycin-stimulation (compare basal condition with oligomycin; Fig. 4g). Although the effect is rather small, this suggests that PI4KIIIβ may partly move away from the endosomes. At first, this redistribution of PI4KIIIβ from endosomes to the cytoplasm seems counterintuitive, since recruitment of this kinase to the membrane is reported as an activation mechanism [44]. A plausible explanation will be discussed. Hence, the data allow to conclude that oligomycin, just like okadaic acid, leads to increased phosphorylation of PI4KIIIβ at Ser294 and binding to 14-3-3, after which this kinase may detach from the (endosomal) membrane.

**Fig. 5:**
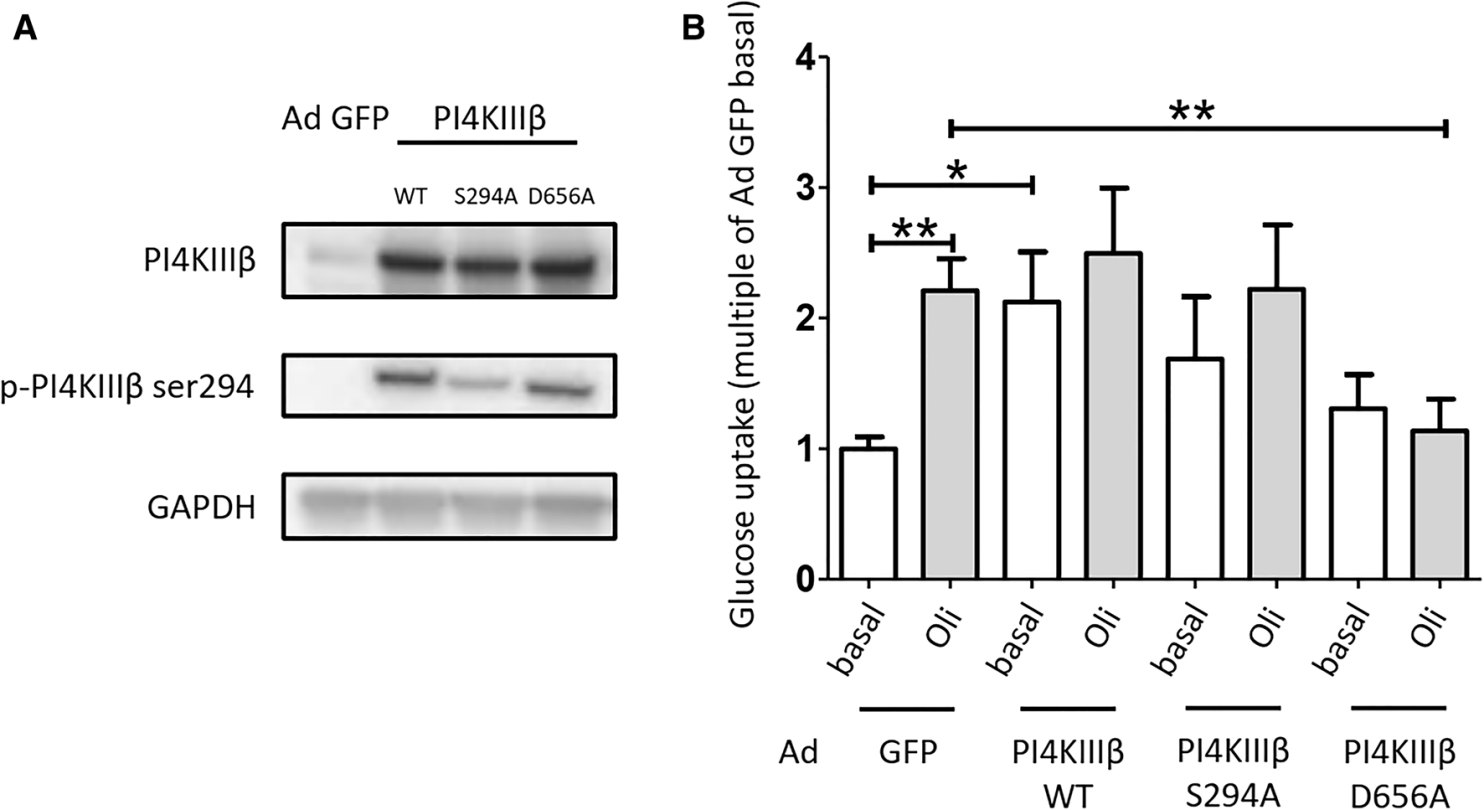
Overexpression of PI4KIIIβ stimulates glucose uptake. Effect of overexpression of PI4KIIIβ wild-type, phosphorylation site mutant (S294A) and kinase-dead mutant (D656A), on basal and oligomycin (Oli)-stimulated glucose uptake in adult rat cardiomyocytes (aRCMs). aRCMs were cultured for 48 h after transduction with adenoviral green fluorescence protein (GFP), PI4KIIIβ wild type, PI4KIIIβ phospho mutant (S294A), and PI4KIIIβ kinase-dead mutant (D656A). **a** A representative western blot of PI4KIIIβ protein expression and PI4KIIIβ phosphorylation. GAPDH was the loading control. **b** Glucose uptake (*n* = 4). aRCMs were treated with or without 5 µM Oli for 15 min. Graphs depict means ± SEM. *P < 0.05, **P < 0.005. Data were normalized to basal

**PI4KIIIβ overexpression stimulates myocellular glucose uptake, dependent on Ser294 phosphorylation and catalytic activity.** aRCMs were cultured for 48 h after transduction with adenoviral PI4KIIIβ wild type (WT), Ser294Ala phosphorylation site mutant (S294A), and Asp656Ala kinase-deficient mutant (D656A). Western blotting was applied to verify the overexpression of PI4KIIIβ variants (Fig. 5a). To obtain further evidence that PI4KIIIβ is a key regulator of contraction-stimulated glucose uptake, we examined the effect of PI4KIIIβ overexpression on glucose uptake in the absence and presence of oligomycin. PI4KIIIβ-WT overexpression enhanced basal glucose uptake, i.e., by 2.2-fold, which is to the same extent as that of oligomycin treatment in mock-transduced cells (Fig. 5b). However, oligomycin did not further stimulate glucose uptake in PI4KIIIβ-WT overexpressing cells, possibly due to saturation effects (with PI4KIIIβ activity level reaching the maximal effect on GLUT4 translocation and glucose uptake rate). In S294A mutant overexpressing cells, basal and oligomycin-stimulated glucose uptake did not significantly change compared to GFP control (Fig. 5b), precluding us from drawing a firm conclusion about the requirement of S294 phosphorylation from this set of data. Importantly, the oligomycin effect was completely abolished in D656A mutant-transduced cells (Fig. 5b). These data suggest that overexpression of PI4KIIIβ-WT augments myocellular glucose uptake and that the PI4KIIIβ-D656A kinase-deficient mutant blocks oligomycin-stimulated glucose uptake, thus confirming the involvement of PI4KIIIβ in glucose uptake triggered by contraction-like signaling.

**Fig. 6:**
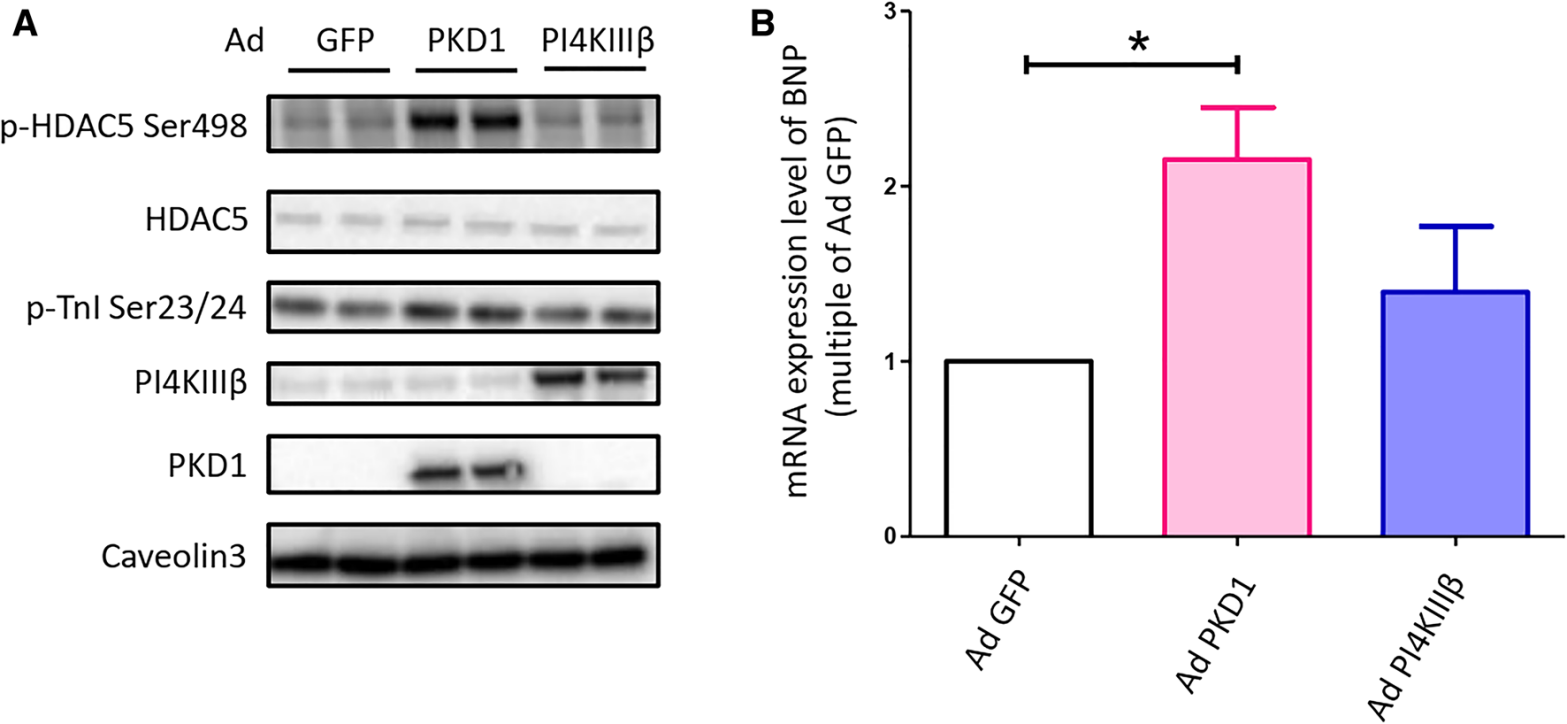
Overexpression of PI4KIIIβ does not increase major hypertrophic biomarkers. Adult rat cardiomyocytes (aRCMs) were cultured for 48 h after transduction with adenovirus (Ad) containing GFP, PKD1, or PI4KIIIβ. **a** A representative Western blotting of protein expression level of p-HDAC5 Ser498, total HDAC5 and p-TnI Ser23/24. Cells were lysed, and western blotting was performed with antibodies against p-HDAC5 Ser498, p-TnI Ser23/24, PI4KIIIβ, PKD1. Caveolin3 was the loading control (*n* = 3). **b** mRNA expression level of BNP. Cells were collected for RNA isolation, and qRT-PCR was performed with BNP primers. GAPDH and cyclophilin A were the house keeping controls (*n* = 3). Graphs depict means ± SEM. *P < 0.05. Data were normalized to Ad GFP

**Unlike PKD1, PI4KIIIβ is not involved in hypertrophic programming of cardiomyocytes.** Besides glucose uptake, PKD1 stimulates hypertrophic growth of cardiomyocytes [23]. Here we examined whether, at the level of PI4KIIIβ, stimulation of glucose uptake can be dissociated from myocellular hypertrophy. Adenoviral overexpression of constitutively active PKD1, which stimulates glucose uptake as reported by us previously [18], also increased phosphorylation of troponin-I and of the hypertrophic repressor histone deacetylase-5 (HDAC5, leading to inhibition of MEF2-dependent hypertrophic programming) (Fig. 6a), both bona fide PKD1 targets [45]. PKD1 expression also upregulates expression of the hypertrophy-associated hormones BNP (Fig. 6b). Unlike PKD1, PI4KIIIβ-WT overexpression did not affect these common hypertrophic markers (Fig. 6a, b). Accordingly, PI4KIIIβ facilitates GLUT4 translocation (Figs. 1, 2, 3) while excluding the major hypertrophic signaling events triggered by PKD1 activation.

**Fig. 7:**
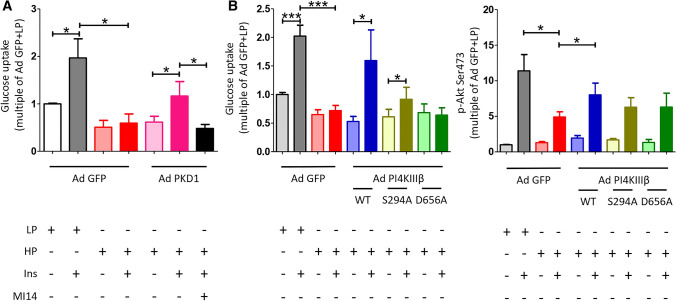
PI4KIIIβ overexpression retains insulin sensitivity during lipid overload. Adult rat cardiomyocytes (aRCMs) were cultured in low palmitate medium (LP), or in medium containing high palmitate (HP) in the presence of adenovirus (Ad) containing either GFP, PI4KIIIβ WT, S294A, D656A, or PKD1. Upon 2 days of culturing, cells were allowed to recover for 30 min prior to short-term (15 min) insulin (Ins, 100 nM) addition with or without MI14. **a** Glucose uptake (*n* = 4), **b** quantification of the level of Akt Ser473 phosphorylation (*n* = 3). Graphs depict means ± SEM. *P < 0.05, ***P < 0.001. Data were normalized to Ad GFP + LP

**PI4KIIIβ protects lipid-overexposed cardiomyocytes from developing insulin resistance.** For examining PI4KIIIβ’s cardioprotective potential in cardiomyocytes during lipid overexposure, we started out with culturing of aRCMs for 48 h with adenoviral PKD1 vector in the presence or absence of MI14, followed by 30 min recovery, short-term (15 min) insulin stimulation, and finally, measurement of glucose uptake. In mock-transduced low palmitate (LP)-treated cells, insulin stimulation enhanced glucose uptake by 2.0-fold. The insulin effect was entirely abolished under high-palmitate (HP) culturing, yet it was restored upon PKD1 overexpression (i.e., 2.0-fold insulin stimulation) (Fig. 7a), in agreement with our previous work [18]. On its turn, this PKD1 effect was abrogated by MI14 (Fig. 7a), indicating that PKD1-mediated preservation of insulin-stimulated glucose uptake in lipid-overexposed cells is due to PI4KIIIβ action. Subsequently, we tested the PI4KIIIβ-WT construct and its mutants on their protective potential in HP-exposed cells. Similar to PKD1 overexpression, PI4KIIIβ-WT overexpression entirely preserved insulin-stimulated glucose uptake (2.0-fold) (Fig. 7b). For comparison, the phosphorylation-site S294A mutant was much less effective and the kinase-dead D656A mutant was unable to maintain insulin-stimulated glucose uptake in HP-exposed cells (Fig. 7b). We also assessed the effects of the three PI4KIIIβ constructs on preservation of insulin signaling in aRCMs during HP culturing, using Akt-Ser473 phosphorylation as readout. In agreement with our previous work [18, 33], HP culturing largely, but not completely inhibited insulin-stimulated Akt-Ser473 phosphorylation. This reduction was partly undone by PI4KIIIβ-WT overexpression, but not by both mutants. In conclusion, kinase activity and Ser294 phosphorylation appear to be crucial for PI4KIIIβ’s ability to protect against lipid-induced insulin resistance.

## Discussion

**Fig. 8:**
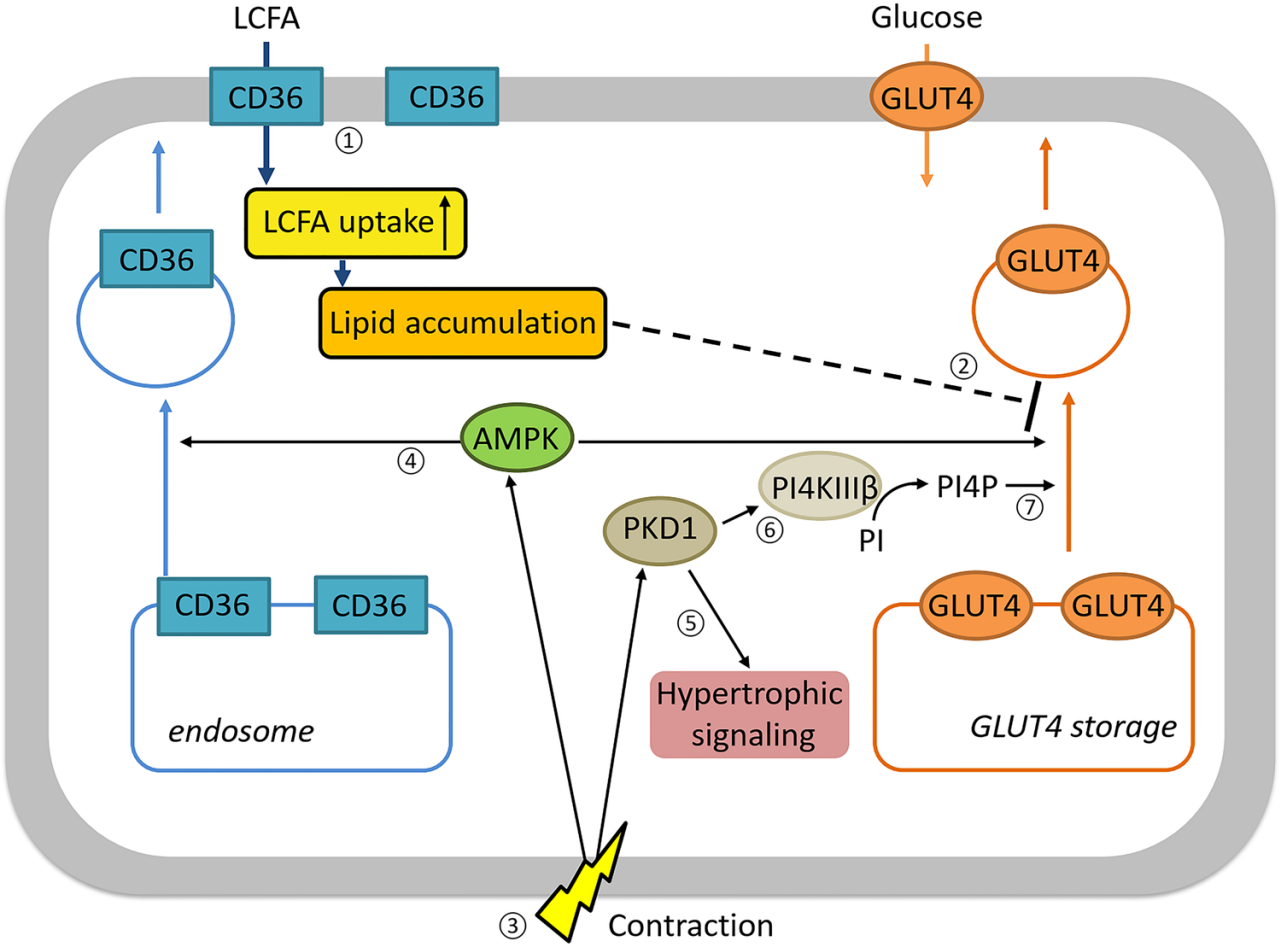
Schematic illustration of the application of PI4KIIIβ as a possible target against lipid-induced insulin resistance via increasing glucose uptake in cardiomyocytes. **①** In the type-2 diabetic heart, there is a permanent CD36 translocation to the sarcolemma, which results in increased LCFA uptake which **②** induces lipid accumulation, leading to the inhibition of insulin-induced GLUT4 translocation and subsequent glucose uptake (a key feature of insulin resistance). **③** Because insulin-induced glucose uptake is deficient in the type 2-diabetic heart, signaling pathways involved in contraction-induced glucose uptake may contain suitable targets to increase cardiac glucose uptake. Contraction activates both AMP-activated protein kinase (AMPK) and protein kinase D1 (PKD1). **④** However, AMPK is also involved in contraction-induced CD36 translocation, while **⑤** PKD1 activates cardiac hypertrophic signaling. **⑥** Therefore, the signaling branch downstream of PKD1 specifically towards contraction-induced GLUT4 translocation would offer superior targets, such as phosphatidyl-inositol-4 kinase-IIIβ (PI4KIIIβ), which is not involved in cardiac hypertrophy. **⑦** PI4KIIIβ regulates contraction-induced GLUT4 translocation via its product PI4P, which may offer a specific platform for accessory proteins involved in formation of GLUT4 vesicles from the endosomes

PI4KIIIβ has been demonstrated to be a bona fide PKD1 substrate [24]. This lipid kinase is present in the trans-Golgi (extending to the endosomes) and is involved in vesicular traffic [24]. One report describes a negative role of PI4KIIIβ in insulin-stimulated GLUT4 translocation in 3T3L1 adipocytes [46]. However, in general, the link between PI4KIIIβ and GLUT4 translocation has not yet been well studied. Moreover, PI4KIIIβ has never been studied in the heart. In the present study, we investigated whether PI4KIIIβ upregulation increases glucose uptake in lipid-overloaded and insulin-resistant cardiomyocytes. Such action is highly desirable in the context of diabetic cardiomyopathy, hinting towards PI4KIIIβ as a possible cardioprotective target (Fig. 8). The obtained results lead us to the following conclusions: (1) PI4KIIIβ is a key component of contraction-induced glucose uptake but is not involved in contraction-induced LCFA uptake. (2) The molecular mechanism of PI4KIIIβ action is related to its kinase activity and PI4P production, but this kinase activity is not enhanced by activation of contraction-like signaling. (3) Unlike PKD1, PI4KIIIβ is not participating in common pathways associated with cardiac hypertrophy. (4) Overexpression of PI4KIIIβ increases basal glucose uptake in cardiomyocytes and preserves insulin sensitivity in lipid-overloaded cardiomyocytes. These aspects are further discussed below.

### Role of PI4KIIIβ in contraction-induced glucose uptake

In this study, we present several lines of evidence that pinpoint PI4KIIIβ as an essential component in contraction-induced glucose uptake. First, contraction/oligomycin-induced glucose uptake is inhibited by MI14. This compound was selected from a series of compounds that have little affinity towards the closely related family member PI4KIIIα, or to the more distant PI4KIIα [40]. We established that MI14 did not inhibit phosphorylation/activation of upstream PKD1 or that of AMPK (situated at the other contraction-activated branch towards GLUT4 translocation), indicating that at least during activation of contraction-like signaling MI14 is a suitable tool to study the involvement of PI4KIIIβ. Second, contraction/oligomycin-induced glucose uptake is inhibited by siRNA mediated silencing, thus providing direct genetic evidence for the requirement of PI4KIIIβ (while avoiding possible off-target effects of the chemical inhibitor MI14). Third, overexpression of PI4KIIIβ-WT stimulates glucose uptake similar to the effect of oligomycin in mock-transduced cells, whereas oligomycin-stimulated glucose uptake is completely blocked by overexpression of PI4KIIIβ-D656A mutant. Collectively, these results strongly suggest a regulatory and possibly rate-governing role of PI4KIIIβ in contraction induced (GLUT4-mediated) glucose uptake, which is compatible with its established function, namely the regulation of trafficking from the Golgi system/endosomes to the plasma membrane [47].

In contrast to glucose uptake, PI4KIIIβ is not involved in contraction-induced LCFA uptake. This restriction to glucose uptake was expected given that PI4KIIIβ’s direct upstream activator PKD1 is not involved in LCFA uptake either [18]. In another perspective, this is an unexpected finding given that contraction-induced LCFA uptake also relies on endosomes, which are not only a storage compartment for GLUT4 but also for CD36. Upon contraction induction, GLUT4 and CD36 both depart from the endosomes, but CD36 translocation apparently does not require PI4KIIIβ-mediated PI4P formation. Perhaps, CD36 translocation is dependent on another lipid kinase producing a different lipid species needed for initiation of budding of CD36-containing vesicles from the endosomes, but this needs further studies. This also allows the speculation that PI4KIIIβ’s presence within the endosomes is restricted to specific sub-compartments, at least those containing GLUT4. Taken together, we identify PI4KIIIβ as a novel signaling component with the ability to push cardiomyocyte substrate utilization towards increased glucose.

### PI4KIIIβ stimulation of glucose uptake requires PI4P formation

Supplying cells with PI4P (the product of PI4KIIIβ) would bypass the need for PI4KIIIβ enzymatic activity and support glucose uptake in MI14-pretreated cells. In pilot experiments, we used diC4-PI4P, which readily permeates biological membranes, but proved ineffective in establishing any effects on glucose uptake at any concentration tested (results not shown). In subsequent experiments underlying Fig. 3, we used diC16-PI4P, which better reflects the naturally occurring PI4P species in cellular membranes [48]. However, this compound needs a carrier system for intracellular delivery. Such carrier (we used a histone-based system) can never be as effective in delivering PI4P specifically at the endosomes compared to the localized production of PI4P by PI4KIIIβ, which could already explain the observed partial protection. Additionally, inefficient delivery and a possible rapid degradation of PI4P species within the 30 min exposure time could limit the ability of exogenous PI4P to simulate endogenous PI4KIIIβ action. Remarkably, the stimulatory effect of PI4P can only be observed upon contraction/oligomycin stimulation in combination with PI4KIIIβ inhibition by MI14, and not by PI4P alone. The likely explanation for this resides in the notion that contraction-induced GLUT4 translocation needs additional activation of AMPK, followed by phosphorylation of AS160/TBC1D1 and recruitment of Rabs for driving the vesicle trafficking step [14]. As explained in the introduction, contraction-induced AMPK activation operates independently of and in parallel to activation of the DAPK–PKD1–PI4KIIIβ axis.

The mechanism by which endosomal production of PI4P facilitates GLUT4 translocation will be the challenge of future investigations. PI4P is known to play an important role in recruitment of a distinct set of proteins to the trans-Golgi, including proteins involved in vesicle budding. The coat protein family of clathrins is of special relevance in this context, as PI4P has been found to stimulate the formation of clathrin-coated vesicles at the trans-Golgi [49]. These vesicles are of fundamental importance in subcellular trafficking of cargo proteins, including GLUT4, i.e., by means of the clathrin heavy chain-22 isoform [50]. Alternatively, there may be other PI4P binding proteins involved in GLUT4 translocation such as CtBP1-S/BARS, a key protein inducing vesicle fission at the Golgi/endosomes [51]. In contrast, PI[4,5]P2 appears not to be involved in contraction-induced glucose uptake. Whereas PI4P is the key phosphoinositide species of the Golgi/endosomes, PI[4,5]P_2_ is more localized to the plasma membrane [47]. Another question to be tackled is the mechanism by which PI4KIIIβ is stimulated by contraction-like signaling to produce more PI4P. We investigated the involvement of phosphorylation at Ser294, and it was indeed found that oligomycin treatment resulted in phosphorylation of this site. Moreover, this phosphorylation is essential for stimulation of glucose uptake since the S294A mutant was ineffective. We also found that oligomycin treatment increased the interaction of PI4KIIIβ with the adaptor protein 14-3-3. This is in line findings in HEK293 cells, which were treated with the toxin okadaic acid, thereby inducing PI4KIIIβ-Ser294 phosphorylation and promoting PI4KIIIβ binding to 14-3-3 [26]. These combined events then appear to increase PI4KIIIβ enzymatic activity and PI4P production in these HEK cells [26]. However, using two different in vitro kinase assays to assess PI4K activity, we did not find any stimulation by oligomycin treatment of cardiomyocytes. Also okadaic acid was ineffective in both assays, suggesting that the effect of Ser294 phosphorylation and 14-3-3 binding on PI4KIIIβ activity is cell-type dependent.

Another mechanism by which contraction-like signaling could induce PI4P production is an alteration in localization of PI4KIIIβ. In this respect type-III PI4Ks have been shown to migrate from their residential cytoplasmic localization to membranes upon overexpression of specific binding partners [44]. Remarkably, we observed an opposite migration pattern upon stimulation of cardiomyocytes with oligomycin or okadaic acid, as evidenced by subcellular fractionation and supported by data obtained from immunofluorescence microscopy (Fig. 4f, g). This suggests that PI4KIIIβ redistributes away from the endosomes into the cytoplasm. Knowing that there is a substantial pool of PI within the cytoplasm bound to PI transfer proteins (PITPs) [52–55], we speculate that upon contraction-like signaling stimulation, PI4KIIIβ may be triggered to interact with these PITPs. This is a reasonable guess since PITPs are known to stimulate PI4P synthesis [56]. To become available for GLUT4 translocation, we expect that this newly formed PI4P is subsequently transferred from PITPs to the Golgi/endosomes. Supportive evidence for this scenario is provided by the observed key role of PITPs in PI4P-mediated build-up of the Golgi in the neocortex [57]. The role of a putative PI4KIIIβ-PITP axis in GLUT4 translocation deserves further studies.

### PI4KIIIβ as a possible target against lipid-induced insulin resistance

As described in the introduction, PKD1 overexpression protects against lipid-induced insulin resistance in the heart, but also induces the pathological condition of hypertrophic growth via HDAC5 phosphorylation. As a downstream component of PKD1 signaling, we hypothesized PI4KIIIβ to be more specifically involved in GLUT4 translocation. Indeed, in contrast to PKD1, PI4KIIIβ is neither involved in phosphorylation of contractile proteins (i.e., TnI [22]) nor in phosphorylation of HDAC5, and accordingly does not appear to induce the hypertrophic program associated with enhanced expression of BNP [58]. Furthermore, the protective effect of PKD1 overexpression on lipid-induced insulin resistance is mediated via PI4KIIIβ, since it was absent in the presence of MI14. The notion that PI4KIIIβ upregulation by itself protects against lipid-induced insulin resistance in the heart, is demonstrated by the observations that PI4KIIIβ overexpression preserved insulin signaling and insulin-induced glucose uptake in lipid-overloaded cardiomyocytes. In contrast, overexpression of the S294A and kinase-dead mutants was unable to fully preserve both processes, indicating that these beneficial actions of PI4KIIIβ are dependent on Ser294 phosphorylation and its kinase activity, respectively. The mechanism behind the beneficial actions of PI4KIIIβ upregulation may include a re-balancing of substrate uptake in the diabetic heart following the enforcement of GLUT4 translocation. Such re-balancing of substrate uptake in cardiac disease is an emerging concept that has been shown to be a viable treatment strategy in many rodent models of different cardiomyopathies [4]. How rebalancing of substrate uptake operates at the detailed molecular level has not yet been fully resolved, but likely Randle cycle effects are involved. In conclusion, we have identified PI4KIIIβ upregulation and addition of PI4Ps as novel treatment options to preserve insulin sensitivity in cardiomyocytes in the face of lipid overload. Future studies could be aimed at identifying the proteins that are docking onto the endosomal PI4P platforms for initiating GLUT4 vesicle budding and translocation, and at investigating whether upregulation of these proteins also could be used as antidiabetic strategies.

### Electronic supplementary material

Below is the link to the electronic supplementary material.

The effect of PI4KIIIβ inhibitor MI14 with different concentrations on oligomycin (Oli)-stimulated glucose uptake in HL-1 cells. HL-1 cells were pretreated with or without 1 µM, 2.5 µM, 5 µM or 10 µM MI14 for 15 min followed by a treatment with or without 1 µM Oli for 30 min. Graphs depict means ± SEM. ***P<0.001. Data were normalized to basal (TIFF 3214 kb)

Efficiency of PI4KIIIβ knockdown via siRNA in HL-1 cardiomyocytes. Cells were cultured for 48 h after transfection with non-coding (n.c.) siRNA or PI4KIIIβ siRNA. On the left: A representative western blot of PI4KIIIβ protein expression. GAPDH was the loading control. On the right: quantification of the level of PI4KIIIβ expression (TIFF 3299 kb)

The effect of PI4KIIIβ inhibitor MI14 on PI4KIIIβ activity. 253 ng PI4KIIIβ (ProQinase) was incubated with or without 2.5 µM MI14 for 10 min. ADP-Glo^TM^ kinase assay from Promega was used to measure PI4KIIIβ activity (TIFF 2405 kb)

## Data Availability

The authors confirm that the data supporting the findings of this study are available within the articles and its supplementary materials. Not applicable.

## Abbreviations

aRCMs: Adult rat cardiomyocytes
LCFA: Long-chain fatty acid
PKD1: Protein kinase D1
PI4KIIIβ: Phosphatidylinositol-4-kinase-IIIβ
PI4P: Phosphatidylinositol-4-phosphate
PI[4,5]P_2_: Phosphatidylinositol-4,5-phosphate
EFS: Electric field stimulation
Oli: Oligomycin
OKA: Okadaic acid
Stau: Staurosporine
Ins: Insulin
LP: Low palmitate
HP: High palmitate
Ad PI4KIIIβ WT: Adenoviral overexpression of PI4KIIIβ wild type
Ad PI4KIIIβ S294A: Adenoviral overexpression of PI4KIIIβ phosphorylation site mutant
Ad PI4KIIIβ D656A: Adenoviral overexpression of PI4KIIIβ kinase-dead mutant
Ad PKD1: Adenoviral overexpression of protein kinase-D1
Ad GFP: Adenovirus-containing green fluorescent protein
IRAP: Insulin-regulated aminopeptidase
